# Case report: Myocarditis in congenital STAT1 gain-of function

**DOI:** 10.3389/fimmu.2023.1095595

**Published:** 2023-03-20

**Authors:** Frederik Staels, Willem Roosens, Simone Giovannozzi, Leen Moens, Jan Bogaert, Cecilia Iglesias-Herrero, Rik Gijsbers, Xavier Bossuyt, Glynis Frans, Adrian Liston, Stephanie Humblet-Baron, Isabelle Meyts, Lucas Van Aelst, Rik Schrijvers

**Affiliations:** ^1^ Department of Microbiology, Immunology and Transplantation, Allergy and Clinical Immunology Research Group, KU Leuven, Leuven, Belgium; ^2^ Department of Microbiology, Immunology and Transplantation, Laboratory of Adaptive Immunology, KU Leuven, Leuven, Belgium; ^3^ Department of Pharmaceutical and Pharmacological Sciences, Laboratory of Molecular Virology and Gene Therapy, KU Leuven, Leuven, Belgium; ^4^ Department of Microbiology, Immunology and Transplantation, Laboratory of Inborn Errors of Immunity, KU Leuven, Leuven, Belgium; ^5^ Department of Imaging and Pathology, Translational MRI, KU Leuven, Leuven, Belgium; ^6^ Department of Microbiology, Immunology and Transplantation, Experimental Laboratory Immunology, KU Leuven, Leuven, Belgium; ^7^ Laboratory of Lymphocyte Signaling and Development, Babraham Institute, Cambridge, United Kingdom; ^8^ Department of Pediatrics, University Hospitals Leuven, Leuven, Belgium; ^9^ Department of Cardiovascular Diseases, University Hospitals Leuven, Leuven, Belgium

**Keywords:** primary immunodeficiencies, STAT1 GOF, myocarditis, infectious susceptibility, chronic mucocutaneous candidiasis

## Abstract

Autosomal dominant Signal transducer and activator of transcription 1 (STAT1) gain-of-function (GOF) mutations result in an inborn error of immunity characterized by chronic mucocutaneous candidiasis, recurrent viral and bacterial infections, and diverse autoimmune manifestations. Current treatment consists of chronic antifungal therapy, antibiotics for concomitant infections, and immunosuppressive therapy in case of autoimmune diseases. More recently, treatment with Janus kinases 1 and 2 (JAK1/2) inhibitors have shown promising yet variable results. We describe a STAT1 GOF patient with an incidental finding of elevated cardiac troponins, leading to a diagnosis of a longstanding, slowly progressive idiopathic myocarditis, attributed to STAT1 GOF. Treatment with a JAK-inhibitor (baricitinib) mitigated cardiac inflammation on MRI but was unable to alter fibrosis, possibly due to the diagnostic and therapeutic delay, which finally led to fatal arrhythmia. Our case illustrates that myocarditis could be part of the heterogeneous disease spectrum of STAT1 GOF. Given the insidious presentation in our case, a low threshold for cardiac evaluation in STAT1 GOF patients seems warranted.

## Introduction

Signal transducer and activator of transcription 1 (STAT1) gain-of-function (GOF) is an inborn error of immunity, affecting more than 400 patients worldwide. Autosomal dominant gain-of-function mutations lead to hyperexpression and hyperactivation of STAT1, which is a major transcription factor playing a pivotal role in regulating a range of biological processes upon cytokine stimulation. It is the most common genetic cause of inherited chronic mucocutaneous candidiasis (CMC), but patients also suffer from recurrent infections and a broad range of autoimmune manifestations (1). Current treatment consists of chronic antifungal therapy, antibiotics for concomitant infections, and immunosuppressive therapy in case of autoimmune diseases. More recently, treatment with Janus kinases 1 and 2 (JAK1/2) inhibitors have shown promising yet variable results (2–5). We describe a STAT1 GOF patient with an incidental finding of elevated cardiac troponins, leading to a diagnosis of a longstanding, slowly progressive idiopathic myocarditis, attributed to STAT1 GOF. Treatment with a JAK-inhibitor (baricitinib) mitigated cardiac inflammation on magnetic resonance imaging (MRI) but was unable to alter fibrosis, possibly due to the diagnostic and therapeutic delay, which finally led to fatal arrhythmia.

## Case description

**Figure 1:**
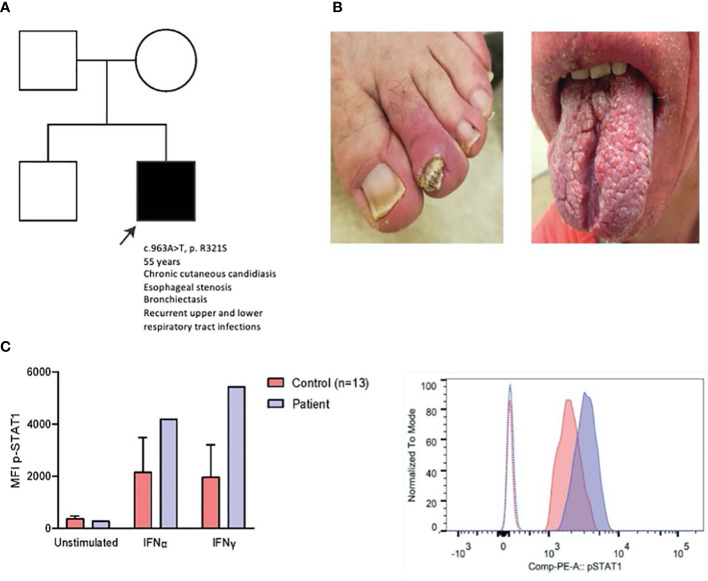
Clinical, genetic and laboratory findings in a patient with STAT1 GOF. **(A)** Patient pedigree. c.963A>T was demonstrated using Sanger sequencing (data not shown), **(B)** Clinical pictures of onychomycosis (left) and impact of CMC on the tongue (right), **(C)** p-STAT1 MFI of patient and age-matched controls (n=13) on CD14^+^ monocytes unstimulated and after IFNα (2000 IU/ml) and IFNγ (2000 IU/ml) stimulation for 15 minutes (left); representative histogram of p-STAT1 on CD14+ monocytes in patient and healthy control (right) after IFN-γ stimulation (15 min). MFI: mean fluorescence index.

We report a case of a 55-year-old man with a *de novo* congenital STAT1 gain-of-function mutation (c963A>T, p.R321S), as previously reported and validated (**Figure 1A**) (1, 6–8). The patient presented with chronic mucocutaneous candidiasis (CMC) (**Figure 1B**), complicated by esophageal stenosis, respiratory infections, and bronchiectasis. He received continuous oral antimycotics since childhood, resulting in transient resistance to ketoconazole, fluconazole and itraconazole (**Supplementary E1**). Genetic and molecular diagnosis (**Figure 1C**) of STAT1 GOF was established at an age of 48 years.

**Figure 2:**
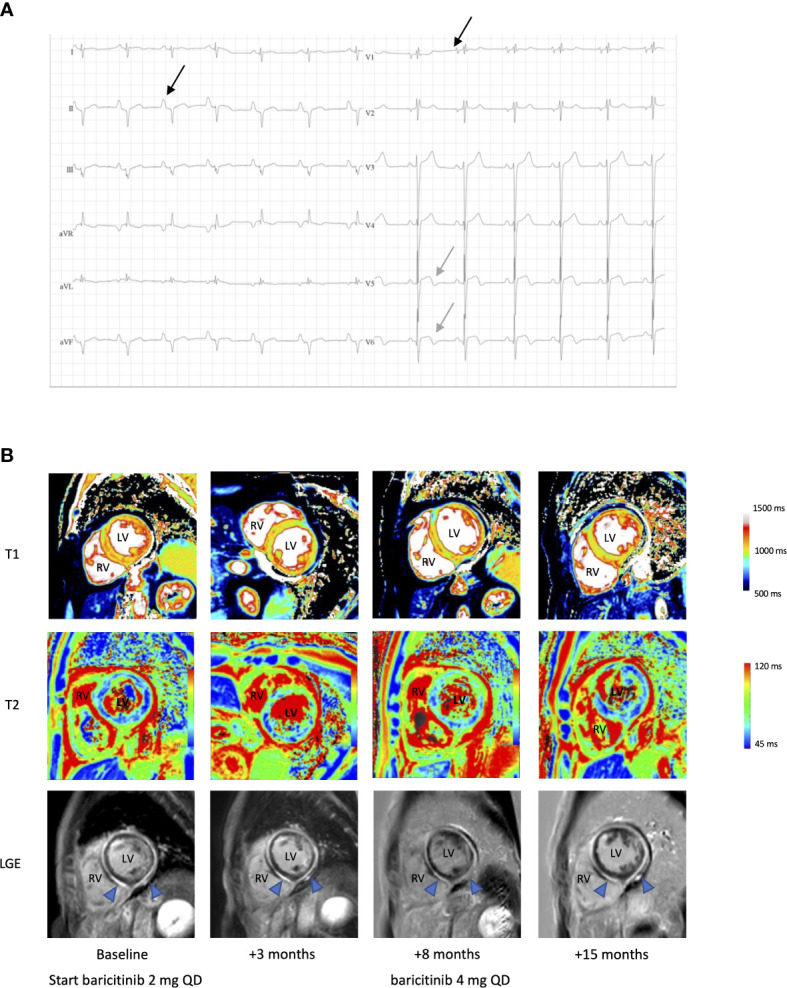
Electrocardiographic and radiological findings in a patient with STAT1 GOF and myocarditis. **(A)** ECG, showing bi-atrial enlargement (black arrow, biphasic P wave in V1 and enlarged P wave in II), extreme rightward QRS axis deviation and biphasic T waves in V5-6 (grey arrow). **(B)** Cardiac native T1 map, T2 map and late gadolinium enhancement (LGE) MR images pre- and post-baricitinib. LV, left ventricle; RV, right ventricle. Blue arrow in LGE showing diffuse enhanced subepicardial and myocardial enhancement compatible with active myocarditis, partial regression after 8 months of treatment, with further reduction at 15 months after a dose increase.

The patient displayed stable respiratory dysfunction. Pulmonary evaluation (**Supplementary E2**) revealed a progressive obstructive pattern and mild diffusion defect. Chest computed tomography (CT) showed bilateral bronchiectasis. Remarkably, high-sensitivity cardiac troponins were elevated on a routine blood sampling at the age of 49, during a hospital admission for infectious enteritis (0.038 mcg/L, reference ≤ 0.013, **Supplementary E2**). No prior measurements were available, yet elevated troponins persisted. The patient did not report thoracic pain or other symptoms suggestive of ischemic heart disease or congestive heart failure. Blood pressure and jugular venous pressure were normal. No peripheral edema was noted. Electrocardiogram (**Figure 2A**) showed a pattern suggestive of right ventricular hypertrophy, biatrial enlargement and biphasic T waves in lateral leads V5-6. Transthoracic echocardiogram (TTE) showed biventricular concentric hypertrophy and mild diastolic dysfunction with increased left ventricular pressures. Cardiac MRI confirmed these findings, with increased native T1 and T2 values and enhancement of the subepicardial myocardium in the middle and apical zone of the left ventricle on late gadolinium enhancement (LGE), suggesting diffuse myocarditis (**Figure 2B**). Holter monitoring, performed in the context of two (pre)syncopal events, showed frequent ventricular extrasystoles and rare runs of non-sustained ventricular tachycardia (VT). Ischemic etiology was deemed unlikely based on CT imaging showing absence of coronary stenotic lesions. There were no clinical or radiological arguments for (chronic) pulmonary embolism as a cause of elevated right ventricular pressure. Also, arrhythmogenic cardiomyopathy was considered unlikely (9, 10). Whole exome sequencing for congenital cardiomyopathy was not performed (11, 12).

There were no arguments for invasive fungal infection based on repeated serum blood cultures, beta-D-glucan and aspergillus antigen measurements (**Supplementary E2**). PCR analysis on blood for common cardiotropic virus, including cytomegalovirus, Epstein-Barr virus, Parvovirus B19 and human herpesvirus-6 and –8 was negative rendering chronic infectious myocarditis improbable. Serology or PCR for other viral origins (enteroviral, adenoviral, human herpesvirus-7 or through microbial cell-free DNA analysis) was not accomplished. Negative autoimmune serology (**Supplementary E2**), together with the absence of other clinical or biochemical criteria, argued against myocarditis in the context of well-defined autoimmune diseases, although STAT1 GOF is associated with an increased likelihood for autoimmunity (1). Finally, drug-induced or hypersensitivity myocarditis was deemed improbable. Therefore, the tentative diagnosis of idiopathic myocarditis with unknown onset, presumably immune-mediated in the context of STAT1 GOF was made. No endomyocardial biopsy (EMB) was performed, considering the inherent risk of the procedure and the low probability of chronic viral induced myocarditis (13). Cardiac transplantation or an implantable cardioverter-defibrillator was refused after a multidisciplinary consult. During follow-up, left ventricular function decreased to a nadir left ventricular ejection fraction (LVEF) of 41% on TTE and 30% on MRI (**Supplementary E2**), with persistently active myocarditis on cardiac MRI. Conventional treatment for heart failure with reduced ejection fraction was initiated, including angiotensin converting enzyme inhibition (perindopril 5 mg QD), diuretic treatment (bumetanide 1 mg QD) and a beta blocker (carvedilol 6.25 mg BID). Initially no clinical deterioration was observed except for one episode of cardiac decompensation triggered by a diverticulitis. Because of active myocarditis with profound effects on systolic function, baricitinib, a JAK1/2 inhibitor, was started at a daily dose of 2 mg. After 3 and 8 months of treatment, partial regression of myocarditis was noted on MRI (**Figure 2B**). The patient reported stable exertional dyspnea and improvement of CMC was noted within 3 months of treatment initiation. During this period, there were no major infectious events or need for additional antibiotic treatment. After 8 months, baricitinib was increased to 4 mg daily, with further reduction of cardiac inflammation on MRI at 15 months. However, myocardial fibrosis already occurred, probably due to longstanding inflammation and resulted in persistent biventricular systolic failure. Unfortunately, shortly thereafter, the patient died from a cardiac arrest precipitated by a witnessed VT. Obduction was not performed.

## Discussion

We report a STAT1 GOF patient with an incidental finding of elevated cardiac troponins due to myocarditis. The presenting symptom was a stable respiratory dysfunction which was initially attributed to recurrent respiratory tract infections and mild abnormalities on pulmonary function tests. Sporadic myocarditis has been associated with inborn errors of immunity (IEI) either in the context of infectious susceptibility or as an autoimmune manifestation (14, 15). Additionally, iatrogenic causes and direct molecular effects of the inborn error could potentially link myocarditis with IEI. However, to our knowledge, myocarditis in the context of STAT1 GOF has not been previously reported. Here, autoimmune myocarditis, as part of the spectrum of autoimmune manifestations in STAT1 GOF, or a myocarditis directly related to STAT1 GOF, was inferred.

Diagnosis was established based on cardiac MRI. This is the preferred diagnostic tool in stable patients with myocarditis as gadolinium contrast enhanced cardiac MRI can identify myocardial edema and fibrosis with high sensitivity (16). ECG findings such as sinus tachycardia with non-specific ST segment and T wave abnormalities can be present but their sensitivity is low (47%) (17). Inverted T waves in the lateral leads were present in our case, prompting a cardiac evaluation.

Treatment of myocarditis is supportive and the use of antiviral or immunomodulatory drugs including intravenous immunoglobulins for viral-induced or autoimmune myocarditis remains debated (16, 18). Interestingly, the use of JAK-inhibitors has shown a beneficial effect in selected cases of immune-mediated myocarditis associated with immune-checkpoint inhibitors (19, 20) and chronic graft versus host disease outside the context of STAT1 GOF (21). As reported in other cases of STAT1 GOF, ruxolitinib and baricitinib, both acting as JAK1/2 inhibitor, show promising results for improving both CMC as well as autoimmune manifestations (2–5). Importantly, not all patients are responsive and in some patients re-initiation after treatment interruption showed reduced efficacy. Moreover, an increased susceptibility to viral infections has been reported in these cases. Although the *in vitro* STAT1 phosphorylation index after stimulation with IFN-alpha and IFN-gamma initially normalized in our patient, considerable variation was observed during follow-up, despite good adherence (**Supplementary E3**). These *in vitro* findings might not serve as an ideal marker for treatment responses as mentioned earlier (4). Until further prospective multicenter data is available to guide the clinical use of this treatment, a careful risk-benefit analysis should be made on a case-by-case basis to evaluate the applicability of JAK inhibition. In this patient, baricitinib was started because of progressing active myocarditis, with increasing risk of end stage heart failure and sudden cardiac death. Treatment did not ameliorate clinical symptoms but led to a regression of active myocarditis on MRI, although biventricular failure persisted due to myocardial fibrosis, leading to fatal arrhythmia. Our patient had limited initial symptoms. Therefore, a low threshold for cardiac evaluation seems warranted in STAT1 GOF patients, especially when symptomatic.

## Data availability statement

The original contributions presented in the study are included in the article/**Supplementary Material**. Further inquiries can be directed to the corresponding author.

## Ethics statement

The studies involving human participants were reviewed and approved by Ethics committee of University Hospitals Leuven (s58466). The patients/participants provided their written informed consent to participate in this study. Written informed consent was obtained from the individual(s) for the publication of any potentially identifiable images or data included in this article.

## Author contributions

RS and LV were involved in the clinical care of the patient. XB and GF were responsible for the laboratory analysis. JB analyzed the MRI images. FS and RS planned the case report and initiated the first draft. All authors contributed to the article and approved the submitted version.
