# Supplementary material for: Case report: Myocarditis in congenital STAT1 gain-of function
Source: Front Immunol. 2023 Mar 20;14:1095595. doi: 10.3389/fimmu.2023.1095595 (PMC10067556; doi:10.3389/fimmu.2023.1095595)
Supplement: Supplementary file 1 [file DataSheet_1.docx]

Supplementary Material

**Supplementary E1**

Summary of the patient’s treatment during follow up.

|  | Dosage | Duration  (time period*) | Reason of cessation if applicable |
| --- | --- | --- | --- |
| **Immunoglobulins** |  |  |  |
| Sandoglobulin | 6 g/month | 1 year  (age 23 to 24) | unknown |
| Ivegam | 30 g/month | 2 years  (age 32 to 34) | normal immunoglobulins – limited infections |
| **Prophylactic antibiotics** |  |  |  |
| Vibramycin | 100 mg QD | 4 years  (age 32 to 36) | insufficient control of respiratory infections |
| Clarithromycin | 500 mg QD | 6 months  (age 40) | intolerance (cough) |
| Doxycycline | 200 mg QD | 1 year  (age 39-40) | insufficient control of respiratory infections |
| Azithromycin | 500 mg three times per week | 13 years  (age 42-55) |  |
| **Antifungal** |  |  |  |
| Amphotericin | IV 450 mg cumulative dose over 10 days  PO 100 mg/ml, 1 ml four times daily | 1 year (age 29)  20 years  (age 34 to 53) | IV stopped because of fever and liver function test abnormalities |
| Itraconazol | 100 mg BID | 29 years  (age 26 to 55) | Intermittent use, continuous since T0 as replacement for ketoconazol |
| Ketoconazol | 200 mg BID | >24 years (until age of 47) | Intermittent use, out of production since T0 |
| Fluconazol | 100 mg TID | 12 years  (age 12 to 24) | Resistance and insufficient disease control, replaced by itraconazol |
| Nystatin | 100,000 IE/mL, 4 to 6 mL four times daily | 6 years  (age 34 to 40) | Sporadically, if uncontrolled CMC |

**Supplementary E2**

Summary of laboratory, functional pulmonary, and cardiac test results during patient’s follow-up.

| **Laboratory values** | Reference values (if applicable) | **T0**  (STAT1 GOF diagnosis at the age of 48 years) | **+2y**  (myo-carditis diagnosis) | **+3y** | **+4y** | **+5y** | **+6y** | **+7y (start baricitinib)** | **+7 y**  **3 months** | **+7 y 8 months** | **+8y** |
| --- | --- | --- | --- | --- | --- | --- | --- | --- | --- | --- | --- |
| WBC | 4-10x10^9^/L | 7.48 | 8.42 | 9.52 | 7.05 | 9.3 | 7.93 | 11.96 | 8.63 | 7.51 | 7.67 |
| CD19^+^ B cells | 82-476/µL | 145 | - | - | - | - | - | - | - | - | - |
| CD3^+^ T cells | 798-2823/µL | 1428 | - | - | - | - | - | - | - | - | - |
| NK cells | 66-745/µL | 200 | - | - | - | - | - | - | - | - | - |
| CRP | ≤ 5 mg/L | - | 5.2 | 8 | 7.1 | 8.3 | 7.3 | 6.7 | 1.4 | 3.4 | 21.6 |
| ANA | Pos/Neg | - | Neg | - | - | - | - | - | - | - | - |
| Farr titer | ≤7 IU/mL | - | < 7 | - | - | - | - | - | - | - | - |
| ANCA | Pos/Neg | - | Neg | - | - | - | - | - | - | - | - |
| Cardiac sarcolemma antibody | Pos/Neg | - | Neg | - | - | - | - | - | - | - | - |
| Cardiac muscle fiber antibody | Pos/Neg | - | Neg | - | - | - | - | - | - | - | - |
| Rheumatoid factor | < 20 IU/mL | - | Neg | - | - | - | - | - | - | - | - |
| Anti-cyclic citrullinated peptide | <7 U/mL | - | 0.7 | - | - | - | - | - | - | - | - |
| Kininase II | 8-52 | - | 21 | 6 | - | - | - | - | - | - | - |
| Beta-D-glucan | < 31pg/mL | - | - | Neg | Neg | - | - | - | Neg | - | - |
| Cryptococcal antigen | Pos/Neg | - | - | - | Neg | - | - | - | - | - | - |
| Aspergillus antigen | Pos/Neg | - | - | Neg | Neg | - | - | - | Neg | - | - |
| EBV PCR | Pos/Neg | - | - | - | - | - | Neg | - | - | - | - |
| CMV PCR | Pos/Neg | - | - | - | - | - | Neg | - | - | - | - |
| Parvovirus B19 PCR | Pos/Neg | - | - | - | - | - | Neg | - | - | - | - |
| HHV type 6 PCR | Pos/Neg | - | - | - | - | - | Neg | - | - | - | - |
| HHV type 8 PCR | Pos/Neg | - | - | - | - | - | Neg | - | - | - | - |
| Blood cultures | Pos/Neg | - | - | - | - | - | Neg | - | - | - | - |
| Troponin T high sensitive | ≤ 0.013 µg/L | - | 0.029 | 0.027 | 0.031 | 0.023 | - | 0.027 | 0.04 | 0.04 | 0.05 |
| Creatine kinase | ≤ 190 U/L | - | 102 | 74 | - | - | 88 | - | - | - | - |
| NT-proBNP | ≤ 172 ng/L | - | 1028 | - | 1096 | - | - | - | 4568 | - | - |
| Lactate dehydrogenase | 135-250 U/L | - | 263 | 229 | 238 | - | 224 | 241 | 248 | 252 | 524* |
| IgG | 7.51-15.6 g/L | - | - | 12.10 | - | - | 13.5 | 11.90 | - | - | - |
| IgM | 0.82-4.53 g/L | - | - | - | - | - | 1.18 | 1.44 | - | - | - |
| IgA | 0.82-4.53 g/L | - | 2.57 | - | - | - | 2.41 | 2.43 | - | - | - |
| Fungal culture | Pos/Neg | - | - | - | Aspergillus sydowii (nail)  Candida albicans (oral plaque) | - | Neg (blood) | - | - | - | - |
| **Lung function** |  |  |  |  |  |  |  |  |  |  |  |
| FEV1s | 3.84 L (predicted) | - | 2.7 | - | 2.6 | 2.11 | 2.32 | - | - | 2.01 | - |
| Tiffeneau index | 78.26% (predicted) | - | 63 | - | 63.62 | 48.68 | 57.92 | - | - | 53.78 | - |
| Total lung capacity | 7.38 L (predicted) | - | 6.45 | - | 6.71 | 6.6 | 6.32 | - | - | 6.10 | - |
| Residual volume | 2.24 L (predicted) | - | 1.99 | - | 2.53 | 2.27 | 2.32 | - | - | 2.74 | - |
| TLCO | 10.79 mmol/min/Kpa (predicted) | - | 9.19 | - | 7.82 | 7.28 | 6.17 | - | - | 5.81 | - |
| **Transthoracic echocardiography** |  |  |  |  |  |  |  |  |  |  |  |
| LVEF (%) | >55% | - | 52 | - | 49 | 47 | 41 | - | - | 32 | - |
| LA sys (mm) | 31.0–47.5 | - | 28 | - | 31.8 | - | - | - | - |  | - |
| LV sys (mm) | 24–38.5 | - | 23.4 | - | 31.3 | 29.1 | 40.8 | - | - | 39.1 | - |
| IVS dias (mm) | 8.62 ± 1.28 | - | 12.1 | - | 12.7 | 9 | 10.3 | - | - | 11.7 | - |
| LV dias (mm) | 40–57.5 | - | 40.7 | - | 42.9 | 38.3 | 46.2 | - | - | 49.9 | - |
| **Cardiac MRI** |  |  |  |  |  |  |  |  |  |  |  |
| Myocardial enhancement on T1 and T2 images |  | - | + (T1 and T2): active myocarditis | + (T1): non active | + (T1 and T2): active myocarditis |  | + (T1 and T2): active myocarditis |  | Regression of myocardial oedema/inflammation | Persistent apical oedema | Regression of myocardial oedema/inflammation |
| LVEF (%) |  | - | 44 | 45 | 36 |  | 30 | 32 | 38 | 39 | 32 |
| RVEF (%) |  | - | 56 | 42 | 38 |  | 20 | 26 | 32 | 25 | 27 |

Laboratory, functional pulmonary, and cardiac test results at diagnosis of STAT1 GOF (T0) and diagnosis of myocarditis (+2 years after molecular diagnosis). Six-year follow up is shown thereafter. A hyphen (-) indicates no testing was performed. Abnormal values are indicated in red. * probable hemolytic sample with falsely elevated LDH.

Abbreviations: WBC: white blood cells; CRP: C-reactive protein; Pos: positive; Neg: negative; FEV1s : forced expiratory volume in 1 second; TLCO: transfer Factor of the Lung for Carbon Monoxide; LA: left atrial; LV: left ventricular; IVS: interventricular septum; LVEF: Left ventricular ejection fraction; Sys: systolic; Dias: diastolic.

**Supplementary E3**

Evolution of STAT-1 phosphorylation index (pSTAT1 index) during treatment with baricitinib.


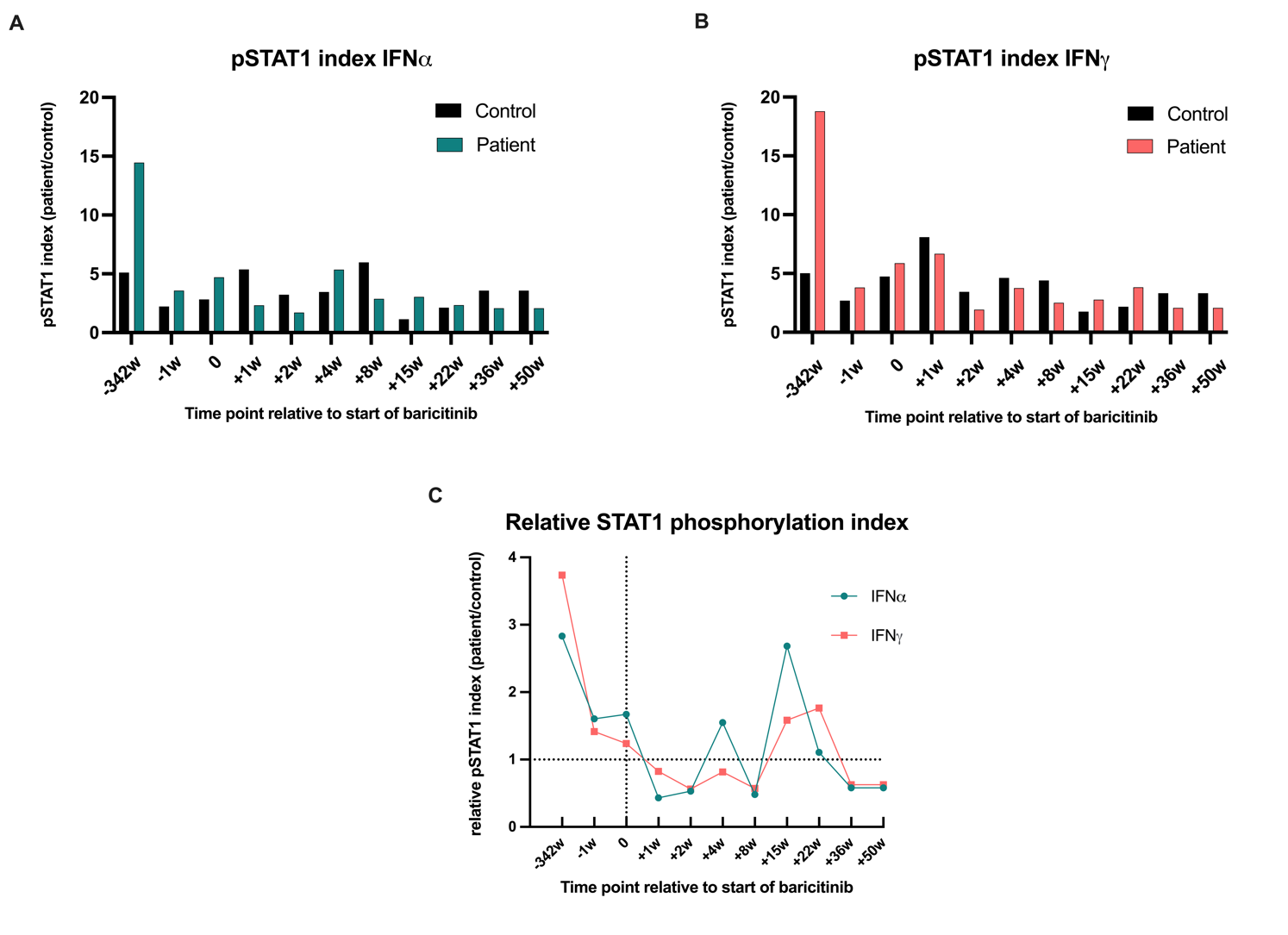


pSTAT1 index of patient and control* (n=1), calculated from MFI on CD14+ monocytes unstimulated and after (**A**) IFN alpha (2000 IU/ml) and (**B**) IFN gamma (2000 IU/ml) stimulation for 15 minutes, measured on different time points during follow-up **(C)** Relative pSTAT index compared to a control patient* and evolution during treatment (pSTAT1 index of patient/pSTAT1 index of control).

*a different control patient was used at every time point and was randomly chosen from a group of ambulatory patients that had blood drawn in our hospital at that day for other reasons, excluding patients from the hematology, oncology, transplantation or pediatric outpatient clinic.

MFI = mean fluorescence index.

**Supplementary methods**

Analysis of phosphorylated STAT1 by flow cytometry

Peripheral blood cells were stained with CD14 APC and CD45 PerCP [BD]. Afterwards, cells were stimulated with IFN-gamma (15 min, 2000 IU/ml), IFN-alpha (15 min, 2000 IU/ml) or left unstimulated. Cells were lysed (Lyse/Fix Buffer [BD phosphoflow]) for 10 minutes at 37°C and washed with 1 mL of Stain Buffer [BD pharmingen]. Thereafter, the cells were permeabilized (Perm Buffer III [BD phosphoflow]) for 30 min on ice. The cells were washed, resuspended in 100 µl stain buffer and stained with PE-labeled antibody specific for phosphorylated STAT1 (pY701) (BD). Phosphorylated STAT1 was evaluated in the CD14+ gate. Analysis was performed on a FACSCanto II instrument (BD).
